# Soluble CD163, a Product of Monocyte/Macrophage Activation, Is Inversely Associated with Haemoglobin Levels in Placental Malaria

**DOI:** 10.1371/journal.pone.0064127

**Published:** 2013-05-22

**Authors:** Caroline Lin Lin Chua, Graham V. Brown, John A. Hamilton, Malcolm E. Molyneux, Stephen J. Rogerson, Philippe Boeuf

**Affiliations:** 1 Department of Medicine (Royal Melbourne Hospital), The University of Melbourne, Parkville, Victoria, Australia; 2 The Nossal Institute for Global Health, The University of Melbourne, Parkville, Victoria, Australia; 3 Malawi-Liverpool Wellcome Trust Clinical Research Programme, College of Medicine, Blantyre, Malawi; 4 School of Tropical Medicine, University of Liverpool, Liverpool, United Kingdom; 5 Victorian Infectious Diseases Service (Royal Melbourne Hospital), Parkville, Victoria, Australia; Institut de Recherche pour le Développement, France

## Abstract

In *Plasmodium falciparum* malaria, activation of monocytes and macrophages (monocytes/macrophages) can result in the production of various inflammatory mediators that contribute to immunopathology. Soluble CD163 (sCD163) is a specific marker of monocyte/macrophage activation typically found at increased levels during various inflammatory conditions and can be associated with poor clinical outcomes. To better understand the relationships between levels of sCD163 and clinical parameters in women with placental malaria, we measured plasma sCD163 levels in maternal peripheral and placental blood compartments at delivery and determined their correlations with birth weight and maternal haemoglobin concentrations. sCD163 levels were negatively correlated with birth weight only in the placental compartment (r = −0.145, p = 0.03) and were inversely correlated with maternal haemoglobin concentrations, both in peripheral blood (r = −0.238, p = 0.0004) and in placental blood (r = −0.259, p = 0.0001). These inverse relationships suggest a potential role for monocyte/macrophage activation in the pathogenesis of malaria in pregnancy, particularly in relation to malaria-associated anaemia.

## Introduction

Malaria infection during pregnancy can be associated with maternal anaemia and low birth weight, which leads to significant disease burden in many populations [1]. A common manifestation of malaria in pregnancy is the sequestration of *Plasmodium falciparum*-infected erythrocytes in placental intervillous blood spaces, a defining feature of placental malaria (PM). PM can initiate a local inflammation (intervillositis or IV), characterised by the accumulation of mononuclear phagocytes (monocytes/macrophages) in placental intervillous blood spaces. Increased numbers of monocytes/macrophages in malaria-infected placentas have been associated with higher prevalence and significantly elevated risk of maternal anaemia and low birth weight [2]–[4]. The latter may be due to a role of PM-associated IV in foetal growth restriction, in which it has been linked to reduced maternal and fetal blood levels of insulin-like growth factor-I [5] and to dysregulated amino acid transport across the placenta [6].

Soluble mediators associated with monocyte/macrophage activation are commonly found at increased levels in malaria-infected pregnant women [7], [8]. High levels of placental interferon-γ, a potent activator of monocytes/macrophages, were associated with low birth weight in Kenyan women [9] but not in a Malawian cohort [10]. Macrophage migration inhibitory factor, which helps to sustain macrophage activation and is implicated in the pathogenesis of anaemia [11], was increased in the placental intervillous blood of women with PM [12]. Monocytes/macrophages constitute a major source of tumor necrosis factor-α, high levels of which in placental blood were associated with severe maternal anaemia [9] and low birth weight [9], [10]. Collectively, these studies suggest that increased monocyte/macrophage activation may contribute to the pathogenesis of PM-associated maternal anaemia and low birth weight.

Soluble CD163 (sCD163) is a novel marker of monocyte/macrophage activation. It is derived from the cleavage of membrane-bound CD163, a monocyte/macrophage receptor involved in scavenging haptoglobin-haemoglobin complexes [13]. Shedding of membrane-bound CD163 is a physiological process, with detectable levels found in normal healthy individuals [14]. In the presence of infection or inflammation associated with monocyte/macrophage activation, this shedding process can be significantly enhanced [15]. High blood levels of sCD163 have been reported in various inflammatory conditions including rheumatoid arthritis [16], coronary atherosclerosis [17] and sepsis [18]. Increased sCD163 levels are often associated with poor clinical outcomes [19], [20]. The relationship between malaria infection and sCD163 is unclear: one study found elevated sCD163 levels in children with uncomplicated malaria compared to severe malaria [21], while in another study where most patients had *P. vivax* infections, sCD163 levels were higher in patients with symptomatic malaria than in uninfected or asymptomatic patients [22].

To better understand the relationship between sCD163 levels and PM pathogenesis, we investigated whether increased sCD163 levels in maternal systemic circulation and in the placenta, correlated with clinical outcomes in PM. We found elevated sCD163 levels in the placental compartment of women with PM and IV compared to uninfected women or to women with PM without IV. There was a weak inverse correlation between placental sCD163 levels and birth weight, while sCD163 levels in both maternal and placental blood negatively correlated with maternal haemoglobin concentrations.

## Materials and Methods

### Ethics Statement

Ethics approval for this study was granted by the College of Medicine Research Committee, University of Malawi and the Melbourne Health Human Research Ethics Committee. Written informed consent was obtained from all participating women.

### Study Samples

Plasma samples were obtained from a previously described cohort [23]. Briefly, participants were pregnant women who attended the Gogo Chatinkha Banda Maternity Unit at the Queen Elizabeth Central Hospital in Blantyre, Malawi from 2001–2006. At delivery, the placenta was cut at the maternal side and blood welling into the incision was collected. Placental and peripheral bloods were both collected into citrate-coated tubes and plasma samples were stored at −80°C. Demographic and obstetric information, including maternal haemoglobin levels at delivery and birth weight, were recorded. Placental villous tissue biopsies fixed in 10% neutral-buffered formalin and embedded in paraffin were used for histologic assessment for malaria as previously described [4].

### Participant Selection Criteria and Malaria Diagnosis

Women in their first and second pregnancies are most susceptible to PM and the associated poor outcomes [24]. Therefore, all primi- and secundigravidae whose paired peripheral and placental plasma samples were available were selected for the current study. Women were categorised into uninfected, PM without IV and PM with IV based on placental histology, as previously described in [4]. Briefly, women with detectable *Plasmodium falciparum*-infected erythrocytes in the placental intervillous blood spaces, in the absence of monocyte accumulation (<5% of all intervillous cells), were classified under “PM without IV” group. Participants with both infected erythrocytes and ≥5% monocyte count in the intervillous blood spaces were classified under the “PM with IV” group. Uninfected women had undetectable parasitaemia in both peripheral blood smear and placental histology sections; these women also did not have monocyte accumulation in the placenta.

### Quantification of sCD163 Levels

Plasma concentrations of sCD163 were measured using a commercial ELISA kit (R&D Systems) according to the manufacturer’s instructions. Capture antibodies prepared at 2 µg/mL were used to coat 96-well plates (Nunc) overnight (100 µL per well). The plates were then washed three times in PBS-Tween20 (0.05%) and blocked with 300 µL reagent diluent (1% BSA in PBS) for an hour. Plasma samples were spun at 1,000×g, 4°C for 15 minutes to pellet debris. Most supernatants were then diluted 1∶100 in reagent diluent; a few samples were diluted up to 1∶500 so that the resultant concentration fell within the range of the standard curve. Standards for sCD163 were prepared by 1∶1 serial dilution, resulting in a 7-point standard curve ranging from 10,000 pg/mL to 156.25 pg/mL. Each sample or standard was added in duplicate after the blocking step and incubated for 2 hours. The plates were washed three times in PBS-Tween20 (0.05%) and detection antibody was added for 2 hours (1 µg/mL; 100 µL per well). After three washes in PBS-Tween20 (0.05%), horseradish peroxidase was added for 20 minutes (100 µL per well). After a final series of washes (thrice in PBS-Tween20 (0.05%)), horseradish peroxidase substrate solution (BD Biosciences) was added for 20 minutes. The colorimetric reaction was then stopped by the addition of 2N H_2_SO_4_ (50 µL per well). Optical density was read at 450 nm with a 550 nm reference wavelength using a microplate reader (Bio-Rad). All incubations were done at room temperature.

### Statistical Analysis

Data were analysed in GraphPad Prism (Version 5.0). The distribution of data was analysed for normality using Shapiro-Wilk test. For non-normally distributed variables, 2-group comparison was performed using Mann-Whitney test. When comparing three groups or more, Kruskal-Wallis test was used and whenever p-value was less than 0.05, a post Dunn’s multiple comparison test was performed. For normally-distributed variables, t-test was used for comparison across two groups, while one-way ANOVA was used for comparing three groups or more. For the latter, a Tukey’s multiple comparison test was performed whenever p-value was less than 0.05. Chi-square test was used to test the difference in the proportions of women with anaemia or low birth weight between all groups. Pearson’s correlation test was used to identify potential correlations between the levels of sCD163 and clinical parameters. In the figures, p-value of less than 0.05 is denoted with *, p<0.01 with ** and p<0.001 with ***.

## Results

### Clinical Characteristics of Study Cohort

Clinical characteristics of the 301 participating women recorded at delivery are summarised in Table 1. Maternal age, haemoglobin levels, parasitaemia and monocyte density were significantly different across groups. Maternal age was significantly lower in the PM with IV group compared to uninfected women (p<0.05). Maternal haemoglobin levels were also significantly lower in the PM with IV group, compared to uninfected women (p<0.01) and PM without IV group (p<0.001). Other parameters including gravidity, gestational age and birth weight did not differ amongst the different groups. The proportion of anaemic women (haemoglobin level <11 g/dL) in each group was significantly different (p = 0.0005), but the proportion of women who delivered low birth weight babies (<2,500 g at time of delivery) was similar across groups (p = 0.2).

**Table 1:** Participants’ characteristics.

	Uninfected	PM without IV	PM with IV	p-value
n	84	147	70	N/A
Maternal age (years)	19 (18, 21)	19 (18, 21)	18.5 (17, 20)^a^	0.03
Gravidity	1 (1, 2)	1 (1, 1)	1 (1, 1)	0.1
Gestational age (weeks)	38 (37, 40)	39 (38–40)	38 (38, 40)	0.09
Haemoglobin concentration (g/dL )	11.9±1.8	12.1±1.9	10.9±2.2^b^	0.0002
Birth weight (kg)	2.94±0.4	2.91±0.4	2.82±0.4	0.2
Number of anaemic cases	24 (28.5%)	40 (27%)	37 (52.8%)	0.0005
Number of low birth weight cases	12 (14%)	17 (11.5%)	14 (20%)	0.2
Monocytes on placental histology (%)	0 (0, 0)	2.2 (1.2, 3)	8.6 (6.3, 12.9)	<0.0001^c^
Parasitised erythrocytes on placental histology (%)	0 (0, 0)	0.6 (0.2, 2)	4 (0.6, 17.9)	<0.0001^d^

Participants were grouped into uninfected women or women with placental malaria (PM), with or without intervillositis (IV), and their characteristics were compared. Data are represented as median (25^th^, 75^th^ percentiles), except for haemoglobin concentration and birth weight (mean ± standard deviation), as well as the number of anaemic and low birth weight cases. The percentages of parasitised erythrocytes and monocytes were determined from 500 cells counted in placental intervillous spaces. All parameters were recorded at delivery. Differences between proportions of anaemic and low birth weight cases were tested using chi-square test. Comparisons for normally distributed and non-normally distributed variables were performed using one-way ANOVA and Kruskal-Wallis test, respectively. When p-value is <0.05, either a post Tukey’s or Dunn’s multiple comparison test was performed and differences that remained significant are as follows:

a p<0.05 for PM with IV group versus uninfected.

b p<0.01 for PM with IV group versus uninfected and p<0.001 for PM with IV group versus PM without IV group.

c, d p<0.001 for uninfected versus both PM and PM with IV groups, and for PM versus PM with IV group.

### Increased Levels of Placental sCD163 in Women with Placental Malaria with Intervillositis

**Figure 1:**
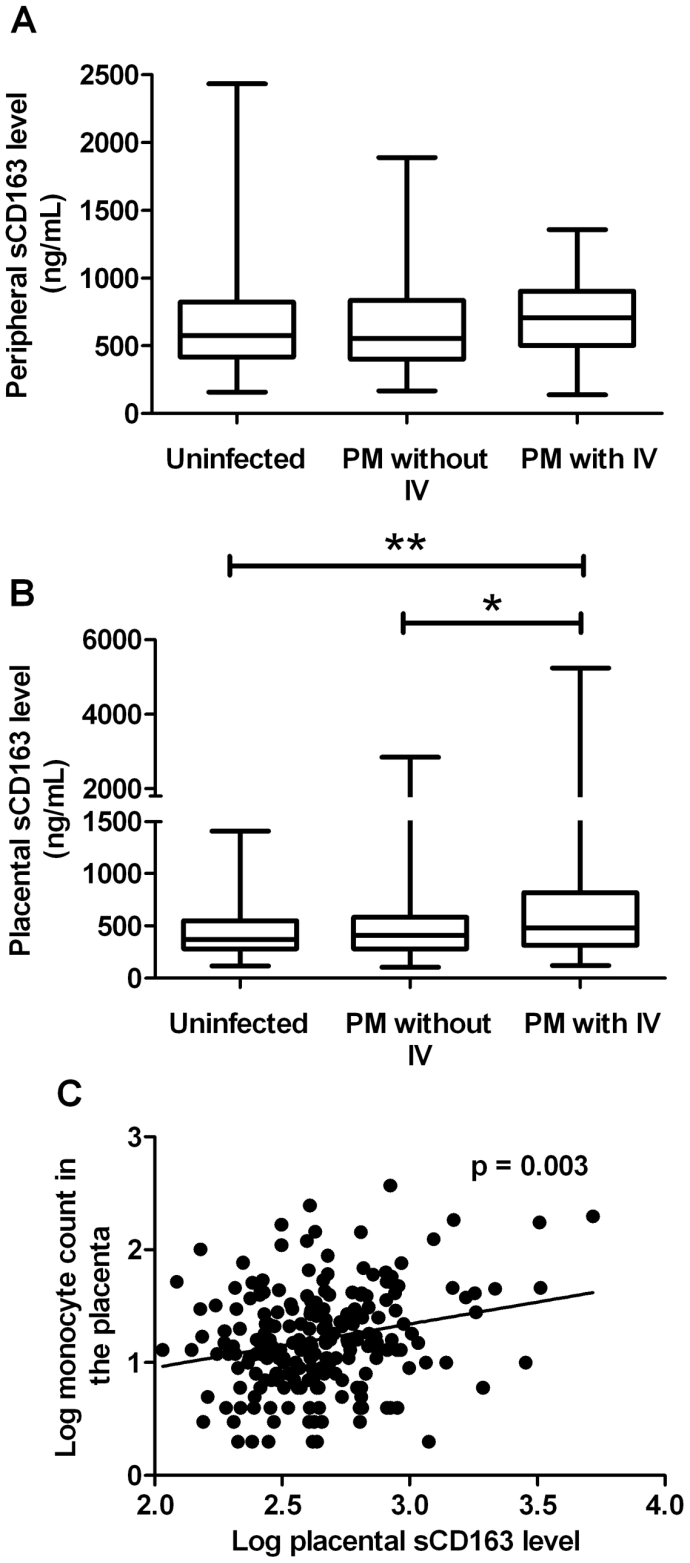
sCD163 levels in peripheral and placental blood. Women were classified into uninfected, placental malaria without intervillositis (PM without IV) or PM with IV groups. (A) sCD163 levels in peripheral blood did not differ between the groups (p = 0.1). (B) Women in the PM with IV group had significantly higher sCD163 placental blood levels compared to uninfected women and women in the PM without IV group (* p<0.05 and ** p<0.01). (C) Monocyte counts in the placenta were positively correlated with sCD163 levels in the placental blood (r = 0.19, p = 0.003).

There was no statistically significant difference in the levels of sCD163 in maternal peripheral blood across groups (p = 0.1) (Figure 1A): [median (25^th^, 75^th^ percentile)] 576.2 ng/mL (417.7, 821.1) for uninfected women, 552.6 ng/mL (400.2, 832.1) for women in the PM without IV group and 705.4 ng/mL (503.5, 900.6) for women in the PM with IV group. In contrast, sCD163 placental blood levels varied significantly across groups (p = 0.0049): the levels were significantly higher in the PM with IV group compared to uninfected women (p<0.01) and women in the PM without IV group (p<0.05) (Figure 1B). For all women with PM (with and without IV), monocyte numbers in the intervillous blood spaces were positively correlated with sCD163 levels in the placental blood (r = 0.19, p = 0.003) (Figure 1C).

### Placental sCD163 Levels and Birth Weight are Negatively Correlated

**Figure 2:**
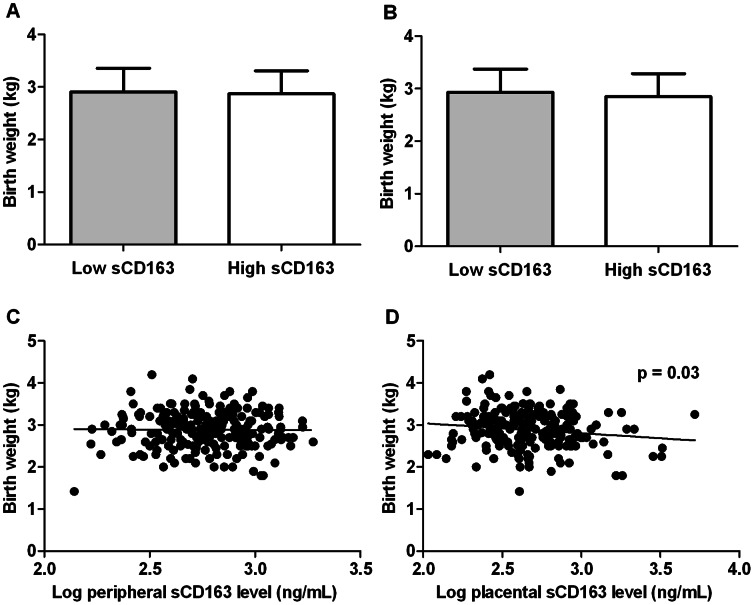
Relationship between sCD163 levels and birth weight. Women with placental malaria were categorized into those with sCD163 levels lower or higher than median levels for each compartment and birth weight was compared across the two groups. (A) Birth weight did not differ between women who had high or low levels of sCD163 either in their peripheral blood (p = 0.5) or (B) in their placental blood (p = 0.1). (C) Birth weight was not correlated with peripheral blood sCD163 levels (p = 0.8) but negatively correlated with (D) placental blood sCD163 levels (r = −0.145, p = 0.03).

To investigate if clinical outcomes were associated with peripheral or placental blood sCD163 levels, infected women were first grouped into those with sCD163 levels higher (high sCD163) or lower (low sCD163) than median levels for each blood compartment. There was no significant difference in birth weight when women were grouped into high or low sCD163, based either on peripheral (p = 0.5) (Figure 2A) or placental blood levels (p = 0.1) (Figure 2B). In addition, peripheral blood sCD163 levels did not correlate with birth weight (p = 0.8) (Figure 2C), but placental sCD163 levels showed a negative correlation with birth weight (r = −0.145, p = 0.03) (Figure 2D). When similar correlation analysis was performed on uninfected women, there was no statistically significant relationship between sCD163 and birth weight in the peripheral (p = 0.6) and placental blood (p = 0.7).

### sCD163 and Maternal Haemoglobin Levels are Inversely Correlated

**Figure 3:**
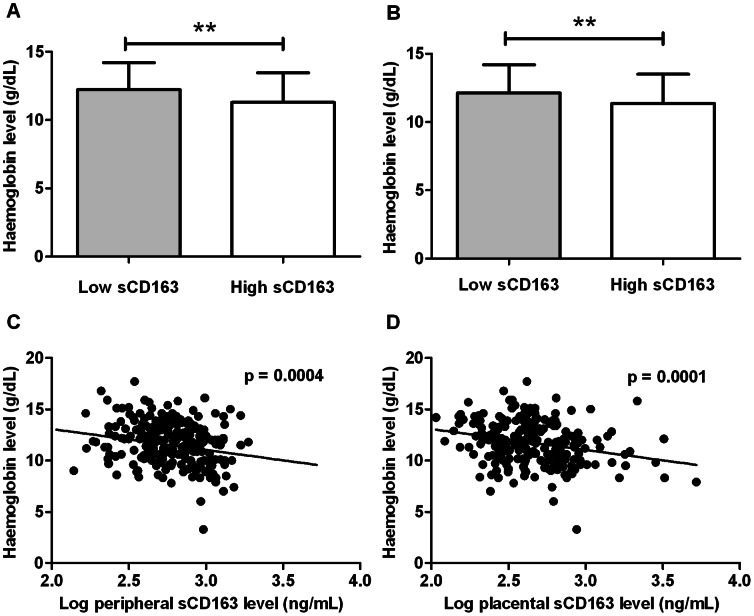
Relationship between sCD163 levels and maternal haemoglobin levels. Women with placental malaria were categorized into those with sCD163 levels lower or higher than median levels for each compartment and maternal haemoglobin levels were compared across the two groups. (A) Women with high peripheral sCD163 levels had significantly lower haemoglobin levels compared to women with low peripheral sCD163 (p = 0.001). (B) A similar relationship was observed for the placental compartment (p = 0.009). Maternal haemoglobin levels correlated negatively with sCD163 levels, (C) in the peripheral blood (r = −0.238, p = 0.0004) and (D) in the placental blood (r = −0.259, p = 0.0001).

Maternal haemoglobin levels were compared between infected women with low and high sCD163 levels (Figure 3). Women with low sCD163 levels in peripheral blood had significantly higher haemoglobin levels (mean ± standard deviation: 12.2±1.9 g/dL) compared to women with high sCD163 levels in peripheral blood (11.3±2.1 g/dL) (p = 0.001; Figure 3A). Similarly, women with low sCD163 in placental blood had higher haemoglobin levels (12.1±2.0 g/dL) than women with high sCD163 levels in placental blood (11.3±2.1 g/dL) (p = 0.009; Figure 3B). Maternal haemoglobin levels were negatively correlated with sCD163 levels in both the peripheral (r = −0.238, p = 0.0004; Figure 3C) and placental blood (r = −0.259, p = 0.0001; Figure 3D). In uninfected women, there was a negative correlation between haemoglobin levels and sCD163 levels in peripheral blood (r = −0.319, p = 0.003), while no statistically significant correlation was observed in the placental blood compartment (p = 0.4). When infected women were grouped into anaemic (<11 g/dL) and non-anaemic (≥11 g/dL) groups, sCD163 levels in both the peripheral (p = 0.007) and placental blood (p = 0.0006) were higher in anaemic women compared to non-anaemic women. sCD163 levels however were not statistically different between anaemic and non-anaemic uninfected women: peripheral blood (p = 0.1) and placental blood (p = 0.2).

## Discussion

Monocyte accumulation in placental blood intervillous spaces during PM is associated with poor clinical outcomes [2]–[4]. In this study, we investigated the relationship between monocyte/macrophage activation, marked by increased sCD163 levels, and clinical outcomes in PM. In a cohort of uninfected women and women with PM, with or without IV, we first compared sCD163 levels by participant group in maternal peripheral and placental blood. We did not observe any differences in the sCD163 levels in maternal peripheral blood across the groups. It was previously reported that maternal peripheral blood monocytes are present in a constant activation state throughout gestation in normal pregnancies [25]. This monocyte activation may have resulted in high peripheral baseline levels of sCD163 in uninfected pregnant women, and reduced a potential difference in sCD163 levels between uninfected women and women with PM, with or without IV. In this connection, it was previously reported that sCD163 levels were elevated in maternal peripheral blood during the first trimester of normal pregnancies [26].

Differences in placental blood levels of sCD163 across the three groups of women were observed. In a previous study, higher sCD163 placental blood levels were detected in women with PM (detected by microscopy) than in uninfected women; that study did not differentiate women with or without IV [27]. In our study, when we separately grouped women with PM into those with or without IV, placental sCD163 was only elevated in women with both PM and IV. We propose that the higher placental sCD163 levels in women with PM and IV can be attributed to both increased monocyte activation and higher number of monocyte counts in their placental intervillous blood spaces. In support of the latter, we found a positive correlation between monocyte numbers in the intervillous blood spaces and sCD163 placental blood levels in infected women.

CD163 is only highly expressed by the classical and intermediate subsets of monocytes [28], suggesting that the high placental blood levels of sCD163 in women with PM and IV could be attributed to selective recruitment and retention of these monocyte subsets in infected placentas. This hypothesis could be further explored by immuno-phenotyping placental monocytes/macrophages.

Monocyte/macrophage activation during malaria infection leads to the production of TNF-α, which at high levels has been repeatedly associated with low birth weight [4], [9]. Although sCD163 levels correlate positively with TNF-α levels [21], we did not find any association between peripheral sCD163 levels and birth weight. There was however, a weak negative correlation between placental blood sCD163 levels and birth weight. Because the biological function of sCD163 is still unclear and we did not measure TNF-α in this study, we cannot ascertain if high sCD163 levels has a direct pathogenetic link to poor birth outcomes or that the inverse correlation is indirectly linked to increased TNF-α levels in the placenta.

In various disease conditions, increased sCD163 levels are usually markers of strong and dysregulated innate immune responses associated with poor clinical outcomes [15]. In malaria, the findings are less conclusive with a study reporting higher sCD163 levels in symptomatic *P. vivax* malaria patients than in asymptomatic or uninfected patients [22], while another study found elevated sCD163 levels in children with uncomplicated malaria compared to those with severe malaria [21]. Increased shedding of CD163 is attributed to monocyte/macrophage activation during inflammation [15], but the resulting sCD163 may have anti-inflammatory properties [15]. Thus, in the latter study, it was hypothesized that malaria patients with higher levels of sCD163 were able to down-regulate inflammation and consequently were protected against severe disease [21]. In the present study, we found higher sCD163 levels in women with PM and IV (the group usually at highest risk of PM-associated outcomes) and in anaemic women. In addition, sCD163 levels were negatively correlated with maternal haemoglobin levels and birth weight. Therefore, our observations suggest that high sCD163 levels are not associated with protection in PM and are more consistent with immune activation rather than down-regulation.

There are several limitations to the current study. Because this was a retrospective study and the women had not been screened for other infectious diseases apart from malaria, we could not rule out this potentially confounding factor. Participant selection based on the availability of paired peripheral and placental blood samples may have introduced selection bias, particularly in the PM with IV group. We propose this because both birth weight and the prevalence of low birth weight were not different in the PM with IV cases compared to the other two groups, unlike previous studies [2], [4]. It is possible that the participants in the PM with IV group in this study may not fully represent the actual burden of IV during PM. Due to the nature of the study, although we found negative correlations between both peripheral and placental blood sCD163 levels and maternal haemoglobin levels for malaria-infected women, we were not able to establish a causative relationship between the two parameters.

Apart from scavenging haemoglobin-haptoglobin complexes, membrane-bound CD163 has also been suggested to bind to erythroblasts and enhance erythroid proliferation [29]. Because sCD163 and membrane-bound CD163 levels are inversely correlated [30], high sCD163 levels may indicate lower membrane CD163 expression and potentially an impairment in the functions mediated by this receptor. Whether or not increased sCD163 levels represent impaired membrane-bound CD163 function in erythropoiesis, leading to lower haemoglobin levels, requires further investigation.

This study is the first to compare peripheral and placental sCD163 levels in uninfected women and in women with PM, with or without IV. Our findings show that increased monocyte/macrophage activation, characterised by high sCD163 levels, is associated with lower haemoglobin levels in malaria infection. Future studies should explore if high sCD163 levels are linked to decreased erythropoiesis, which then contributes to the pathogenesis of malaria-associated anaemia, in pregnant women and potentially in young children.
